# Characterizing the coalescence area of conjoined twins to elucidate congenital disorders in singletons

**DOI:** 10.1002/ca.23725

**Published:** 2021-02-20

**Authors:** Lucas L. Boer, Annelieke N. Schepens‐Franke, Eduard Winter, Roelof‐Jan Oostra

**Affiliations:** ^1^ Department of Imaging Section Anatomy and Museum for Anatomy and Pathology, Radboud University, Medical Center Nijmegen Netherlands; ^2^ Pathologisch‐anatomische Sammlung im Narrenturm‐NHM Vienna Austria; ^3^ Department of Medical Biology, Sections Clinical Anatomy & Embryology, Amsterdam University Medical Centers, Location Academic Medical Center University of Amsterdam Netherlands

**Keywords:** concomitant anomalies, conjoined twins, holoprosencephaly, neural tube defects, OEIS, sirenomelia

## Abstract

Shared anomalies, always located close to the area of coalescence and observable in virtually every type of conjoined twinning, are currently seen as separate anomalies caused by mostly unknown and seemingly unrelated pathways rather than being connected to the twinning mechanism itself. Therefore, most (case) reports about conjoined twins are mere descriptions of (external) dysmorphologies lacking reflections on the possible origin of their concomitant anomalies. As we will demonstrate in this article, shared anomalies are influenced, and in some cases solely and sequentially explained, by interaction aplasia and neo‐axial orientation; two embryological mechanisms to which each set of conjoined twins is subjected and are responsible for their ultimate phenotypical fate. In this review, we consider how the ventral, lateral and caudal conjunction types and their intermediates determine the phenotypic presentation of the twins, including patterns of shared malformations and anomalies, which in themselves can be indistinguishable from those encountered in singleton cases. Hence, it can be hypothesized that certain anomalies in singletons originate in a fashion similar to that in conjoined twins.

## 1 INTRODUCTION

Conjoined twins are still being perceived as results of imperfect monozygotic twinning. However, there are unanswered questions regarding the enigmatic etiopathogenesis of these intriguing entities (Boer, Schepens‐Franke, & Oostra, 2019). Conjoined twins are generally considered rare, though every now and then new (clinical) cases are (scientifically) described (Barnes‐Davis & Cortezzo, 2019). Besides these infrequent manifestations, an important source of information with tremendous potential lies in the hundreds of formalin‐fixed and skeletonized specimens in anatomical museums throughout Europe (Oostra, Baljet, Verbeeten, & Hennekam, 1998). It is in these teratological collections that answers to etiological, pathogenetic and embryologically oriented questions about rare birth defects can be found (Boer, Morava, Klein, Schepens‐Franke, & Oostra, 2017). Throughout many decades or even centuries of collecting, it is inherent in these collections that many specimens of the same type are brought together, making it possible to research and describe rare anomalies without the *N* = 1 problem common in a clinical setting. In addition to their substantial numbers, most of these representatives of the past were collected long before pre‐natal diagnostics became available (Boer, Schepens‐Franke, et al., 2017). This is why these specimens are often near or at full term, revealing unique insights into severe defects at a late stage of development. Severe congenital anomalies (late) in the third trimester are nowadays rare in developed countries, where methods for prenatal assessment of the unborn child are ubiquitous. It is noteworthy that the (scientific) literature concerning conjoined twins often uses the wrong terminology and classification and that etiopathogenetic mechanisms are erroneously interpreted, incomplete, too vague, or just too brief to comprehend and are often incompatible with knowledge about normal embryological development (Bovendeert, Nievelstein, Bleys, & Cleypool, 2020; Spencer, 2003). Moreover, most case reports concern only external dysmorphological aspects. Indubitably, many subtle internal malformation patterns are missed, unknown, or never described in depth, all hindering the search for etiopathogenetic insights into the still unfathomable genesis of conjoined twins. This dearth is even more apparent in conjoined twins showing anomalies located within the boundaries of the conjunction area, and therefore equally divided between and contributed to by both twins (Ozkur, Karaca, Gocmen, Bayram, & Sirikci, 2006). Understandably, owing to their extremely low prevalence, many of these additional anomalies go unnoticed and thereby unreported. In depth descriptions of these types of conjoined twins are therefore rare and are seldom supplemented with valid etiopathogenetic or embryological knowledge. This creates a scientific paradigm in which reflections on our (still) limited knowledge of the etiopathogenesis of conjoined twinning limit us to speculating beyond our current understanding. On the rare occasions when these anomalies are (consciously) observed and reported, they are often perceived and interpreted as concomitant with but causally independent of the conjoined twinning event (Zhang, Yang, & Cui, 2014). This assumption could interfere with the articulation of etiological and pathogenetic models and hamper our reflections on embryologically‐oriented questions concerning the genesis of conjoined twins. However, some of the shared anomalies could be linked to the twinning mechanism itself, especially when they are closely related to the conjunction area. As we will show, these seemingly incidental anomalies are solely and sequentially the results of neo‐axial orientation and interaction aplasia, two embryological adjustments exclusive to conjoined twins (Oostra, Keulen, Jansen, & van Rijn, 2012). Although these two mechanisms are currently discussed only in respect of conjoined twins, it could be hypothesized that they are also present in or influence the ultimate morphology of singletons; multiple causes could potentially generate a heterogeneous outcome. Interestingly, the anomalies described in the present review are described in the current literature as occurring in singletons and correlated with genetic predispositions implicating an ever‐growing multitude of candidate genes. However, to the best of our knowledge, no genetic cause is currently linked to a possible etiology of conjoined twinning, leaving the etiopathogenesis of conjoined twins yet to be elucidated. In this article we review some of the most convincing shared anomalies in symmetrically conjoined twins that could be caused sequentially by the twinning mechanism itself and are, in some cases, indistinguishable from those encountered in singletons. On this basis, we propose a novel approach to explaining and/or investigating relatively common malformations.

## 2 CONJOINED TWINNING: A PHENOTYPIC SPECTRUM DICTATED BY NEO‐AXIAL ORIENTATION AND INTERACTION APLASIA

Although the proposed genesis of conjoined twins has been extensively described in another review (Boer et al., 2019), we will outline it briefly here to introduce and summarize the theories and descriptions of shared anomalies proposed in this review. Two important embryonic adjustments, seen exclusively in conjoined twins, are noteworthy: neo‐axial orientation and interaction aplasia (Oostra et al., 2012). Both mechanisms are responsible for adjustments and alterations of external and internal morphology and determine the ultimate phenotype of the conjoined twins.

**FIGURE 1:**
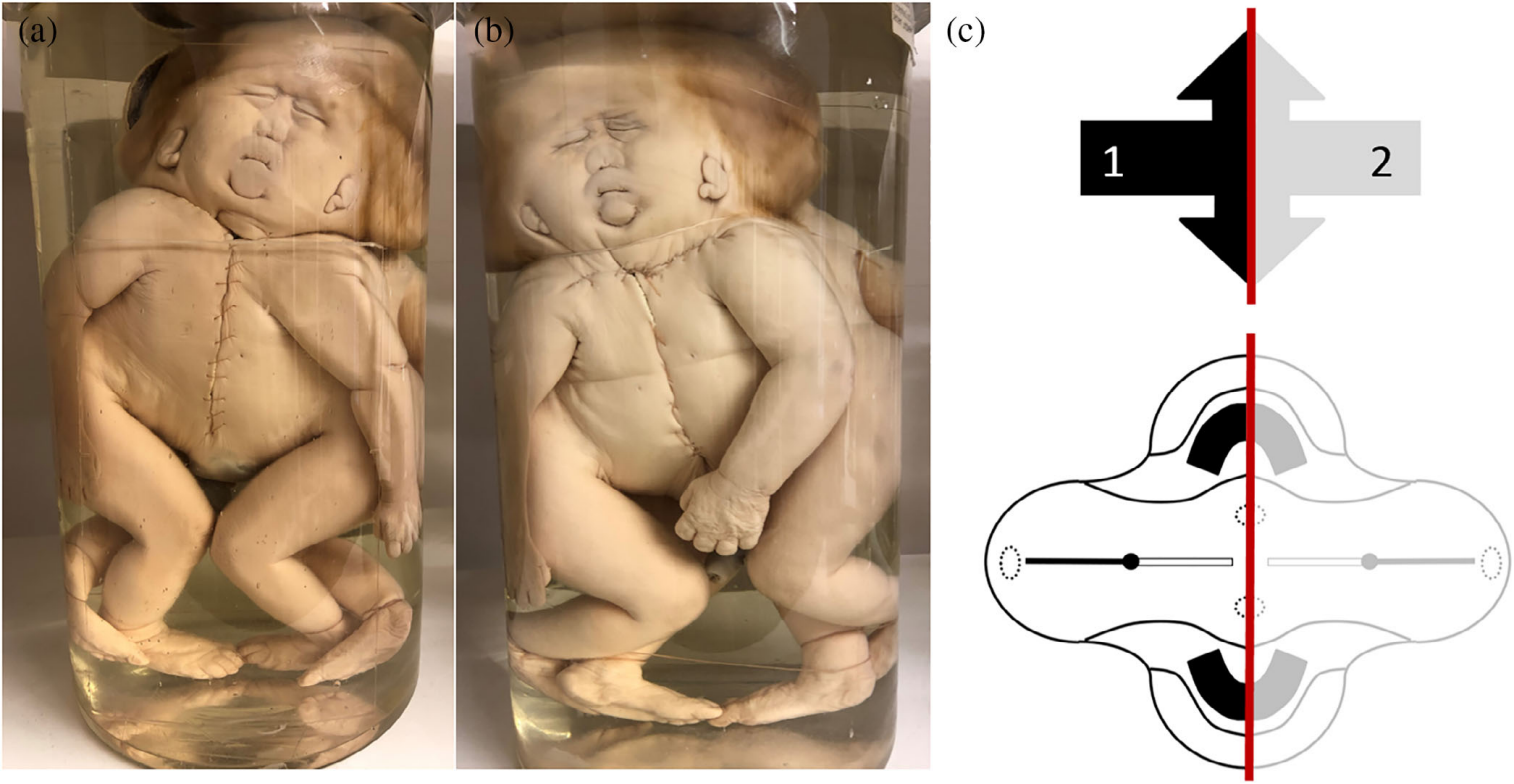
(a) and (b) Severely ventrally united cephalothoracoileopagus twins photographed from both sides with almost perfect symmetry, indicating that the duplicated primordia were in positions nearly opposite to each other. Note that the two faces are compound faces, each twin contributing half of the structures in one face. Specimen from the *Narrenturm* collection in Vienna (Austria). (c) Schematic representation of what profound neo‐axial orientation brings about. As indicated by the black (fetus 1) and gray (fetus 2) arrows, structures belonging to either one of the fetuses are being divided and diverted. This configuration is further delineated in a schematic embryonic disk of a cephalothoracoileopagus seen from its dorsal aspect. The red line indicates the midline of the twins, showing a 50% contribution of each twin over the entire coalescence area

Embryonic disks from ventrally and caudally united twins show duplication of axial structures in opposing configurations and are, with increasing approximation, subjected to neo‐axial orientation (Boer et al., 2019). This embryonic adjustment refers to the mechanism by which opposing homologous structures are divided in the median plane, after which the two halves divert laterally to meet their counterparts from the opposite side. Hence, compound organs and structures are formed by equal contributions from both embryos. The formed structures are located in a plane perpendicular to the original, thereby altering their original topographical location by a 90°axial rotation (Spencer, 2003). From a gross morphological point of view, two more or less normal structures are formed, although each half of these structures originally belongs to one of the twins. Neo‐axial orientation is most dramatically demonstrated in cephalothoracoileopagus twins. These are the most extreme manifestation of ventrally united twins in which two compound faces are formed on each side, in addition to profound thoracic and abdominal conjunctions (Figure 1).

**FIGURE 2:**
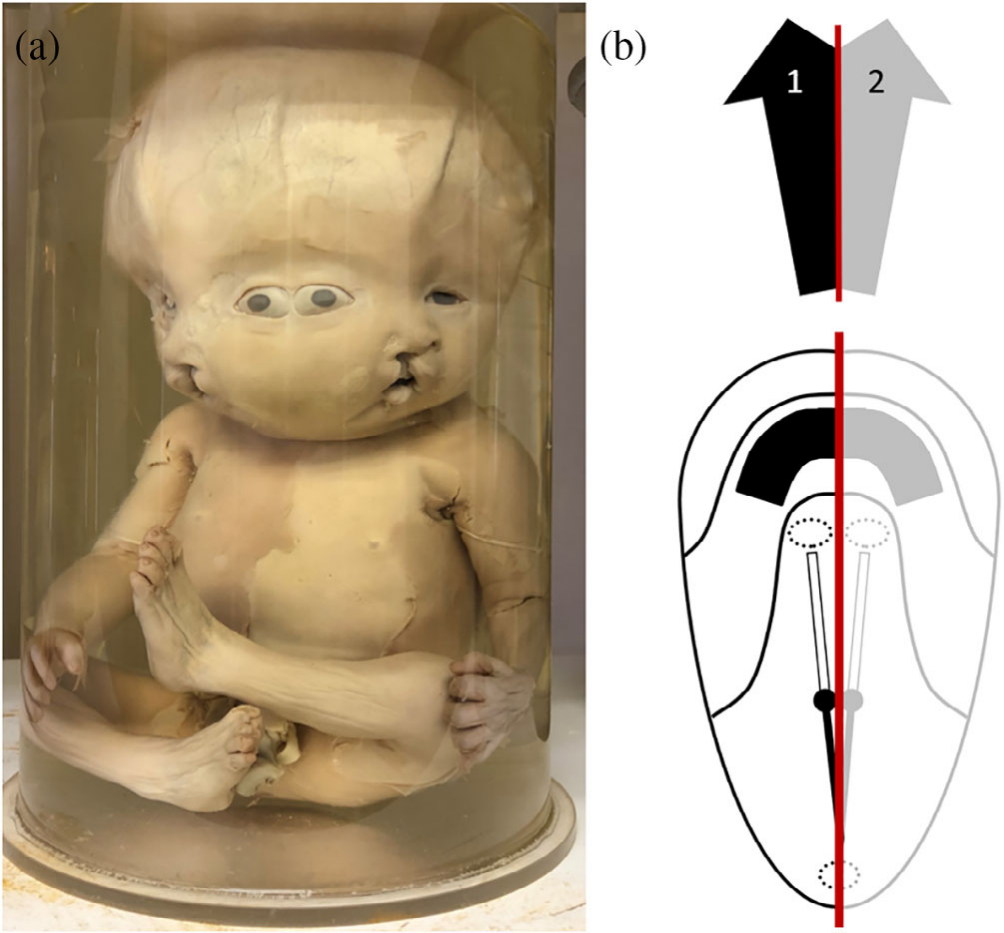
(a) Severely laterally united parapagus diprosopus twins. Note the additional bilateral cleft lip, which is concordant for its severity but discordant for its location (mirror imaged). Specimen from the *Narrenturm* collection in Vienna (Austria). (b) Schematic representation of what profound interaction aplasia brings about. As indicated by the black (fetus 1) and gray (fetus 2) arrows, structures that should be on either side of the midline are absent. This configuration is further delineated in a schematic embryonic disk depicted from the dorsal aspect of a parapagus diprosopus. The red line indicates the midline of the twins; indicating that somewhat more than 50% of each twin is still present

While the opposite positions of the two primordia are responsible for neo‐axial orientation, interaction aplasia sets in when the duplicated primordia have mutual positions other than exactly opposite each other. In interaction aplasia of contiguous primordia, organs and structures in the conjunction area fail to develop (Oostra et al., 2012). The degree of aplasia depends not only on the approximation of the two primordia, but also on their mutual angle (Boer et al., 2019). The closer the approximation and the more acute the angle, the more prominent the interaction aplasia. Suppression of structure and/or organ formation is assumed to result from aberrant concentrations of morphogens in and around the two longitudinal axes and conflicting (molecular) pathways (Levin, Roberts, Holmes, & Tabin, 1996). Primordia become obliterated by these overlapping gradients and consequently fail to form (Machin, 1993). Interaction aplasia is best demonstrated in laterally conjoined parapagus diprosopus twins, the most extreme form of laterally united twins (Figure 2), in which half a body of each twin is not formed except for the axial structures. Both aforementioned mechanisms often occur simultaneously, resulting in intermediate phenotypes, and are then responsible for the formation of certain concomitant anomalies, as discussed below. It is thus essential to appreciate that all laterally, caudally and ventrally united twins share a common embryonic configuration of duplicated central organizers and that all of the resulting phenotypes can be placed in a continuous spectrum of overlapping latero‐caudal and latero‐ventral phenotypes. This spectral model also illustrates a very broad range of phenotypical divergence, implying that each case of conjoined twins is in that respect unique (Boer et al., 2019). Thus, the diversity of external variability depends solely on the mutual position (in both orientation and approximation) of the duplicated embryonic primordia and their subsequent outgrowth. For more background information, the reader is referred to a previous paper on this subject (Boer et al., 2019).

## 3 SHARED, CONCORDANT AND DISCORDANT ANOMALIES

To clarify the principle of shared anomalies in respect of certain types of conjoined twins described in this review, one should be acquainted with general concepts and interpretations of concomitant disorders and descriptions of their location; this, and its actual cause, is often misinterpreted in the literature concerning conjoined twins (Zhang et al., 2014). It is essential to realize that conjoined twinning is a congenital malformation in itself, secondarily influenced by changes related to abnormally united organs and superimposed effects of aberrant hemodynamics due to adjustments after the duplicated primordia have formed (Weber & Sebire, 2010). For the interpretation (and therefore also the examination) of concomitant anomalies in conjoined twins, anomalies located within the borders of the conjunction area (hence by definition “shared”) are to be differentiated from those situated outside those borders.

The shared anomalies can be divided into two groups. The first consists of concomitant anomalies that are unavoidably present. They are directly and inevitably the consequence of neo‐axial orientation and/or interaction aplasia and are present in all conjoined twins of that particular type and degree of conjunction. Examples of anomalies in this first group are duplications, hypoplasias and/or aplasias of limbs, inherently located in the area of coalescence in for instance parapagus, thoracoileopagus and ileoischiopagus twins, or cardiac duplications and hypoplasias in parapagus and thoracoileopagus twins. The same holds for the “holoprosencephaloid” appearance of (usually) one of the compound faces in cephalothoracoileopagus twins. As we will substantiate, this is caused solely by neo‐axial orientation and regional interaction aplasia. This is also the case for the “sirenomeloid” presentation of the compound limb in laterally‐deviated ischiopagus twins.

The second group of shared anomalies does not always arise. These anomalies occur only within particular settings of primordial duplications. Examples are neural tube defects in diprosopus twins, and omphaloceles/OEIS complexes in ventrally united twins (“OEIS” stands for Omphalocele, Exstrophy of the cloaca, Imperforate anus and Spinal defects). Although it is not always obvious that concomitant anomalies originate from the twinning mechanism itself, it can reasonably be assumed that anomalies within the area of conjunction are causally related to the twinning process and are hence influenced by neo‐axial orientation and interaction aplasia. In this review, we present and discuss the abovementioned examples of shared anomalies in symmetrically conjoined twins in detail.

**FIGURE 3:**
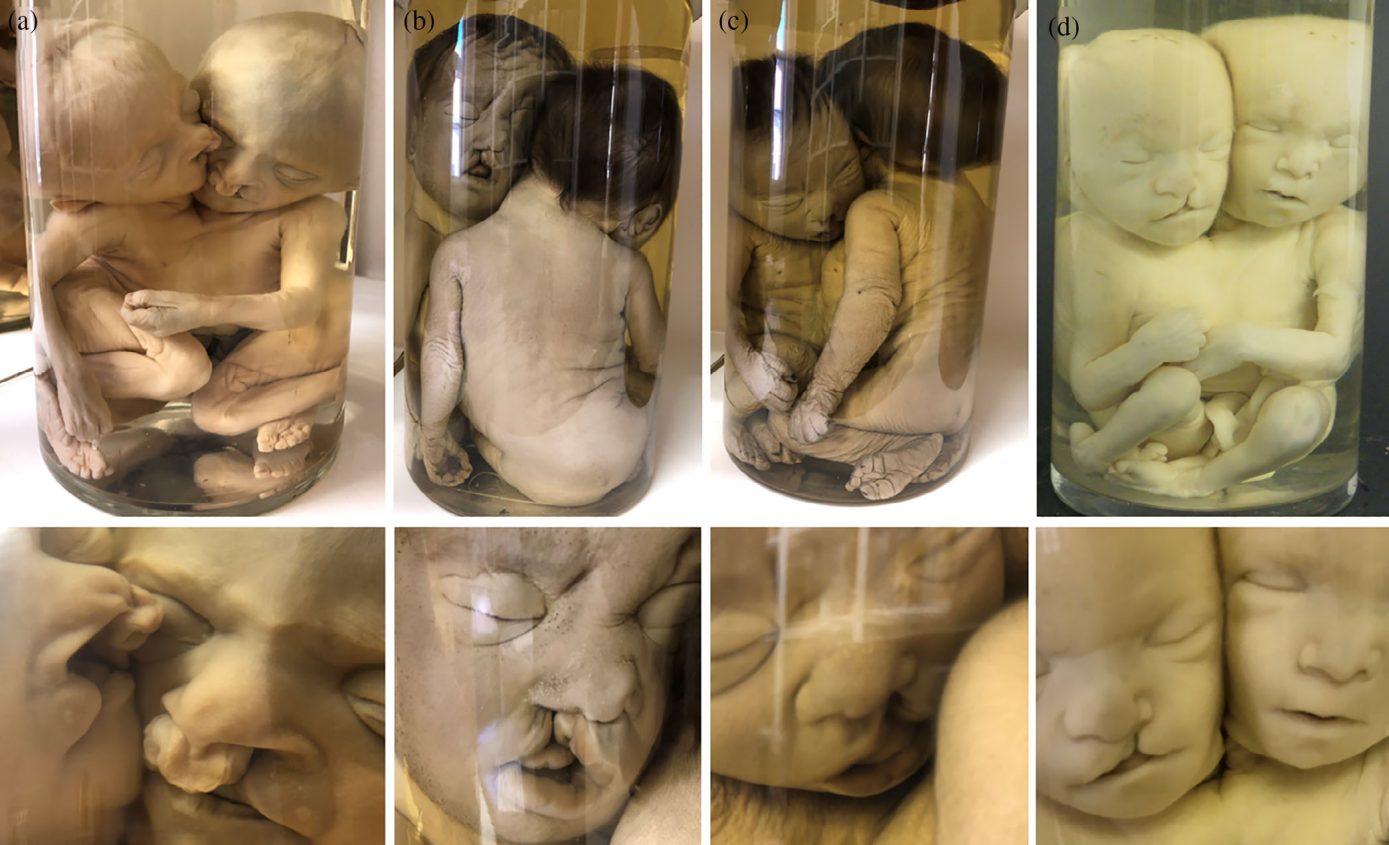
Overview of gross specimens with different types of cleft lip/palate, showing details of each face below each picture. (a) Thoracoileopagus tetrabrachius tetrapus with severe concomitant bilateral concordant cleft lip/palate. Both twins are affected by the same anomaly showing the same degree of severity. (b) and (c) “Ventral” and “dorsal” view of a thoracoileopagus tetrabrachius tetrapus with concomitant concordant cleft lip, which is concordant for severity but discordant for location (mirror imaged). (d) Thoracoileopagus tribrachius tetrapus with concomitant discordant cleft lip; only one member is affected. Specimens from the *Narrenturm* collection in Vienna (Austria)

Furthermore, concomitant anomalies situated outside the borders of the conjunction area are described as concordant and/or discordant with the anomaly, its severity and its location. They can occur in three ways: (a) concordance in both the anomaly and its severity; meaning both twins have the same anomaly with the same degree of severity (Figure 3a); (b) concordance in the anomaly but discordance in its severity or location; both twins have the same anomaly, but its severity and/or location differs between them (Figure 3b,c); and (c) discordance in the anomaly; only one twin presents with the additional anomaly (Figure 3d). These conditions are outside the scope of this paper.

## 4 THE LOWER LIMBS IN LATERALLY‐DEVIATED AND INTERMEDIATELY‐UNITED TWINS

**FIGURE 4:**
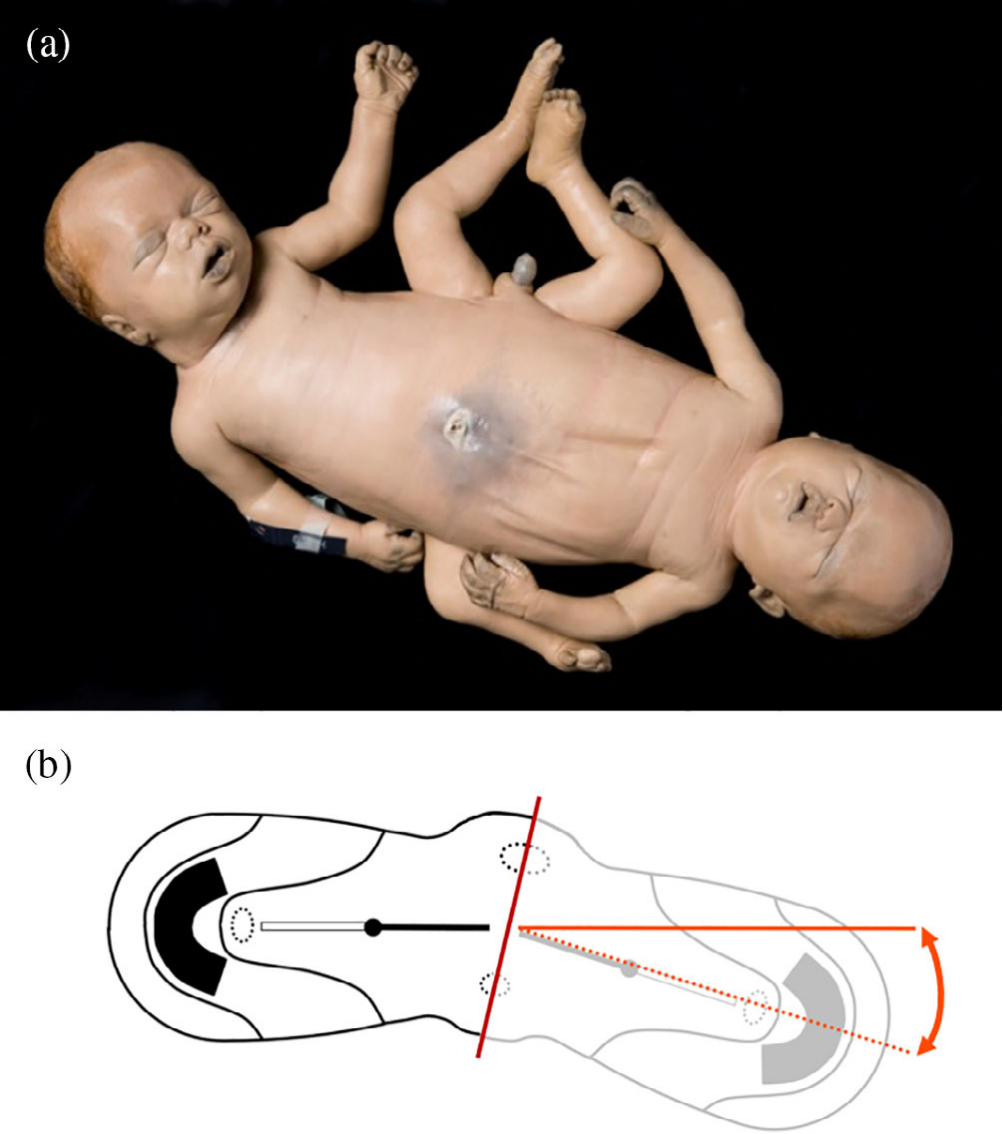
(a) Ilioischiopagus tripus twin with a single shared lower limb on the side affected by regional interaction aplasia. Specimen from the Anatomical Museum in Nijmegen (The Netherlands). (b) Embryonic disk configuration with lateral deviation (red dotted line with angle) resembling the gross anatomy depicted in panel (a)

**FIGURE 5:**
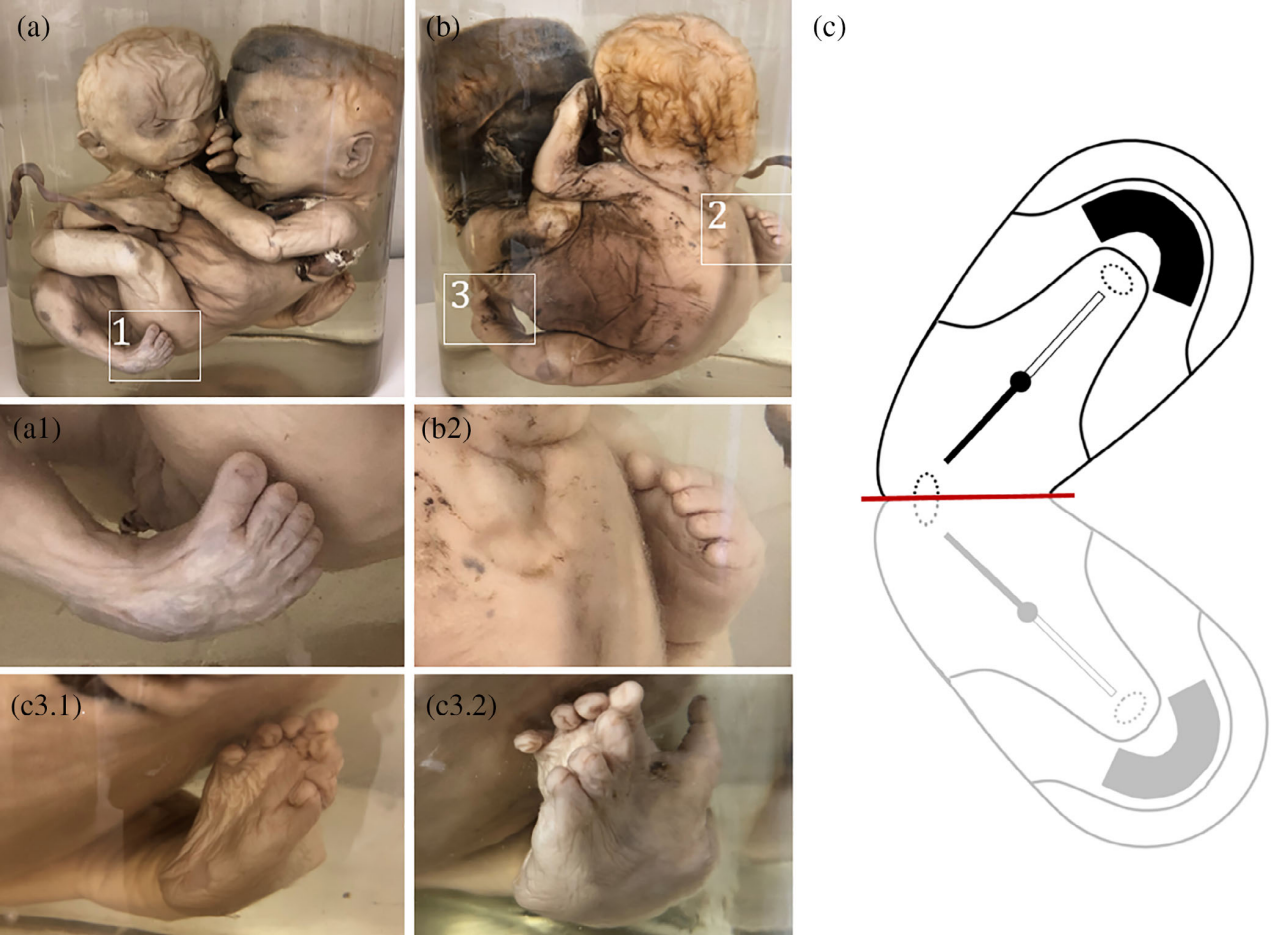
Thoracoileoischiopagus tetrabrachius tripus. (a). Ventral view of the twin with magnified detail of one of the laterally positioned legs with a normally‐formed foot (A1). (b). Dorsal view of the twin with magnified inserts of a second normally‐formed and laterally‐located leg with normal foot (B2) and a shared third, medially‐located leg with multiple toes (C3.1/3.2). Note that the first toe is conjoined and is located dorso‐medially on top of the compound foot. Specimen from the *Narrenturm* collection in Vienna (Austria). (c). Embryonic disk configuration with lateral deviation resembling the gross anatomy depicted in A and B

When ventraly and caudally united twins show lateral deviations (Figure 4), and are therefore affected by interaction aplasia, the mutual distance between opposing limbs at the affected side can be reduced to such a degree that a single shared upper and/or lower compound limb is formed. These are referred to as tribrachius and tripus configurations respectively. In intermediate forms of conjoined twinning, which do not fit with the conventionally defined “groups” and are sometimes referred to as *thoracoileoischiopagi* (Boer et al., 2019; Oostra et al., 1998; Spencer, 2003); tripus with or without tribrachius is a typical characteristic (Figure 5). Lee et al. (1999) extensively described an interesting intermediate tripus type of conjoined twins and noted a dysplastic sacrum, parallel iliac bones and a hypoplastic medially‐located limb with a polydactylous foot. The kidneys and anus were absent and the urinary bladder was indistinct. A very similar condition is found in singletons with sirenomelia. As described extensively elsewhere, the pathogeneses of sirenomelia is still debated and is a subject of ongoing controversies (Boer, Morava, et al., 2017). However, irrespective of the actual cause, it is clear that the malformation of the lower limbs appears at a very early stage of development, or even from the start when the lower limb buds are formed, as the limb buds never rotate medially (Boer, Morava, et al., 2017). As a result the fibulae are located medially and the patellae dorsally. Inherent in this anatomical configuration is that if a compound foot is formed, the first toes are located laterally and the fifth toes (or other toes, with increasing severity of sirenomelia) adjacent to the midline; in consequence, the plantar side of this compound limb faces ventrally (Boer, Morava, et al., 2017). However, despite the general resemblance between the sirenomelic limb in singletons and the tripus limb in conjoined twins, the latter features an essentially different anatomy, in which the first toes not the fifth are adjacent to the midline and therefore unrelated to true sirenomelia.

## 5 FACIAL ANOMALIES IN CEPHALOTHORACOILEOPAGUS TWINS

**FIGURE 6:**
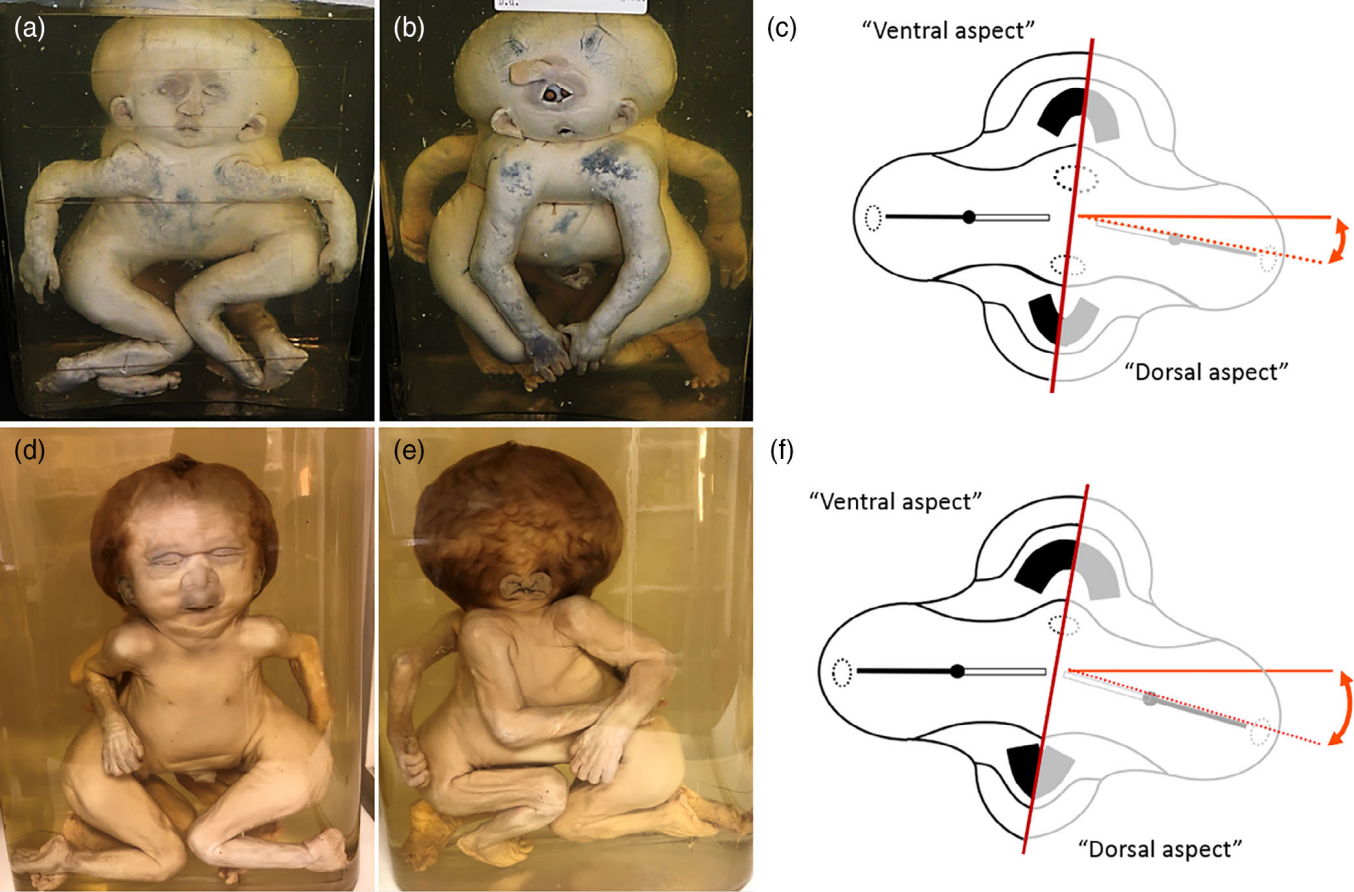
(a) Cephalothoracoileopagus twins; “ventral” view with fairly normal morphology as expected with a neo‐axial affected compound face. (b) “Dorsal” view of the same cephalothoracoileopagus twins as in A with a lateral deviation leading to an apparently holoprosencephalic presentation on the interaction aplasia‐affected side. Specimen from the *Narrenturm* collection in Vienna (Austria). (c) Embryonic disk configuration with lateral deviation (red dotted line with angle) resembling the gross anatomy depicted in panels (a) and (b). The resulting interaction aplasia gives rise to (among other consequences) the holoprosencephalic compound face on the “dorsal” side of the twins. (d) “Ventral” and (e) “dorsal” views of cephalothoracoileopagus twins with an even more pronounced lateral deviation. The “dorsal” side of the twins shows medially‐located set of ears and “vanished” midfacial structures. Specimen from the *Narrenturm* collection in Vienna (Austria). (f) Embryonic disk configuration with lateral deviation (red dotted line with angle) resembling the gross anatomy depicted in panels (d) and (e). This extensive interaction aplasia causes absence of a (compound) oropharyngeal membrane and results in agnathia and aprosopia with absence of all facial structures

**FIGURE 7:**
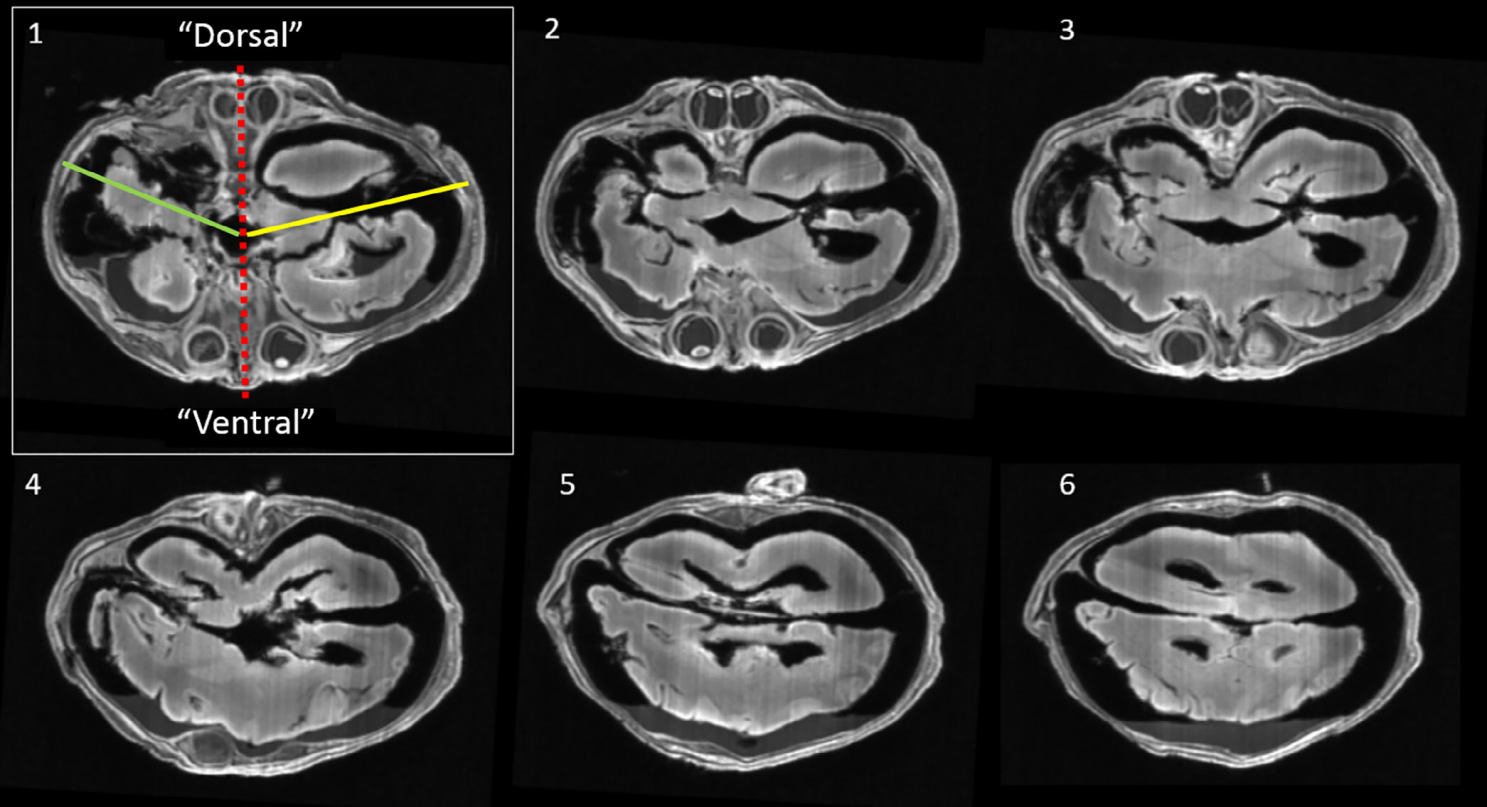
Transverse T1‐weighted magnetic resonance images of the cranial region of the laterally‐deviated cephalothoracoileopagus depicted in Figure 9. Image numbers indicate caudal (1) to cranial (6) positions respectively. Image 1 is accompanied by a red dotted anterior to posterior axis, indicating the midline location of both the divided and diverted facial primordia of each twin, hence dividing the “right“ from the “left” twin. The yellow line indicates the midline of the “left” divided and diverted brain, the green line that of the “right” divided and diverted brain. Clearly noticeable in image 1 is that the smallest angle between the two brains is oriented to the holoprosencephalic or “posterior” face. Note that the frontal lobes, anterior horns of the lateral ventricles and the third ventricles of each twin are conjoined. Unfortunately, the brain is somewhat macerated, potentially interfering with its interpretation. However, and interestingly, in contrast to the facial appearance, the compound brain does not in our opinion show the typical aspects of holoprosencephaly

Symmetrical cephalothoracoileopagus twins are considered to be formed by duplicated embryonic primordia located exactly opposite each other, and their phenotypic appearance is profoundly influenced by neo‐axial orientation (Boer et al., 2019). In addition to thoracic and abdominal conjunction, this results in two complete compound faces on either side of a single united head (Figure 1). However, more often than not, these cephalothoracoileopagi show facial dysmorphologies in one of the compound faces such as microstomia, micrognathia, a hypoplastic nose, hypotelorism and median clefts of the upper lip (Baron et al., 1990) (Figure 6a). These malformations are supposed to result from deviation of the embryonic primordia from their opposing position, leading to interaction aplasia in the compound structures at one side of the conjunction area (often dubbed the “posterior” side). Phenotypically, this results in midline hypoplasias that resemble the complete arhinencephaly/holoprosencephaly spectrum (Boer et al., 2019) (Figure 6b,c), ranging from midfacial hypoplasia and premaxillary agenesis up to cebocephaly (presence of two separate eyes set close together and a small single‐nostril flat nose), ethmocephaly (presence of a blind‐ending appendage called a proboscis separating a narrow set of small eyes with an absent nose), cyclopia, and even aprosopia (Cirstoiu et al., 2016; Oostra et al., 1998) (Figure 6d,e,f). Interestingly, despite the facial resemblance to holoprosencephaly, the typical malformations of the brain seem to be absent (Figure 7).

## 6 OMPHALOCELE AND OEIS COMPLEX IN VENTRALLY AND CAUDALLY UNITED TWINS

**FIGURE 8:**
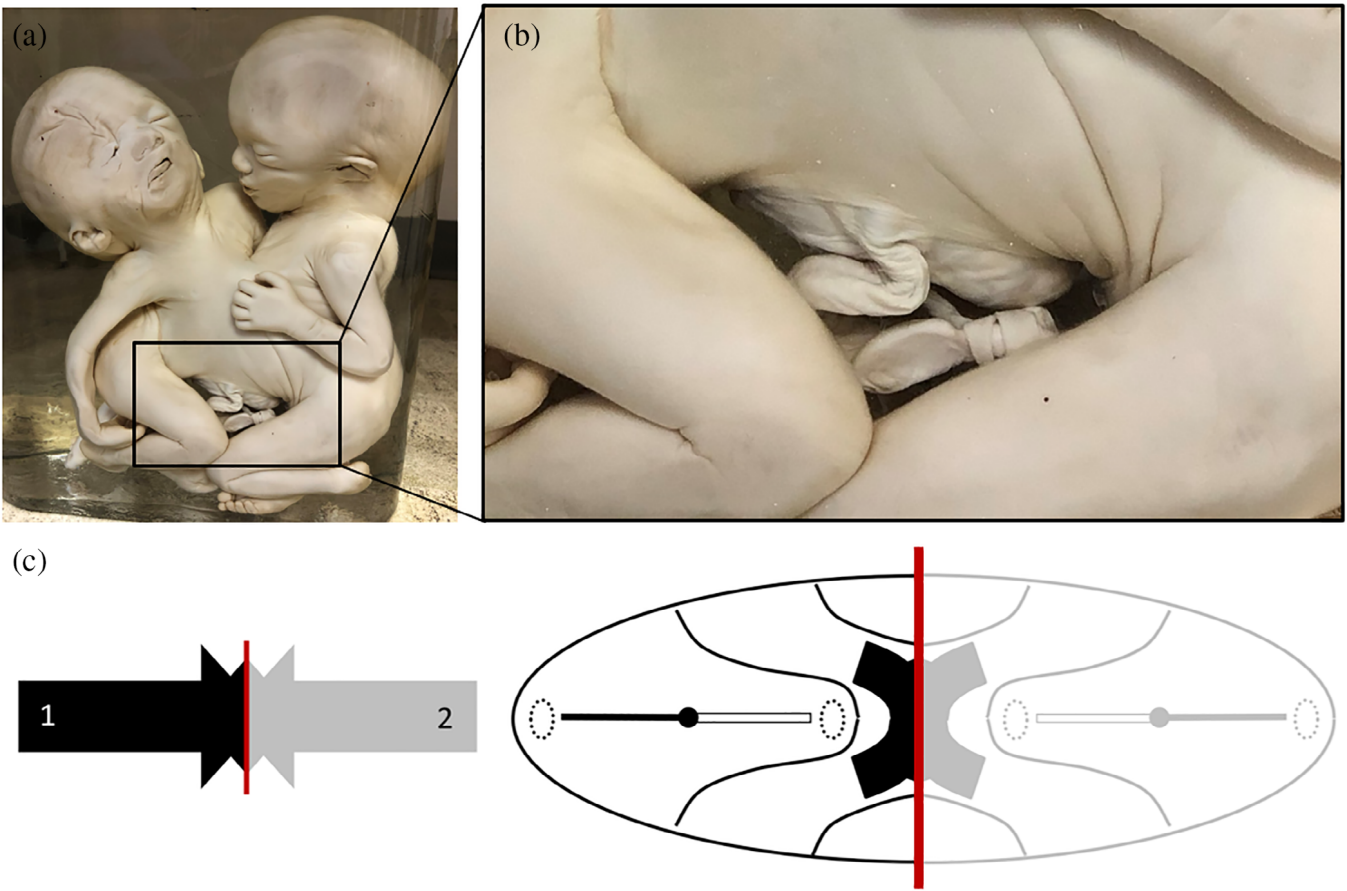
(a) Thoracoileopagus twins with a shared omphalocele suspended between the two members. (b) Clearly noticeable is that a single umbilical cord arises from the amniotic‐lined sac, implying the presence of a single umbilical ring during embryological development. Specimen from the *Narrenturm* collection in Vienna (Austria). (c) Schematic representation of the process when there is relatively mild neo‐axial orientation, indicated by black (fetus 1) and gray (fetus 2) arrows. This configuration is further delineated in a schematic embryonic disk from the dorsal aspect of a thoracoileopagus tetrapus. The red line indicates the midline of the twins, clearly showing that medially located structures (in this example heart, liver, diaphragm and the ventral body wall) will be shared. Note the broad circumferential umbilical ring (the outer contour of the embryonic disk). It is conceivable that when embryological growth succeeds the cranio‐caudal and lateral folding processes, the complex duplicated embryonic disk is incapable of closing completely at the future abdominal wall, ultimately interfering with caudal developmental patterns. Furthermore, opposing midgut structures could potentially be incapable of retracting inside the abdomen and hence become passively “locked” in the median plane

**FIGURE 9:**
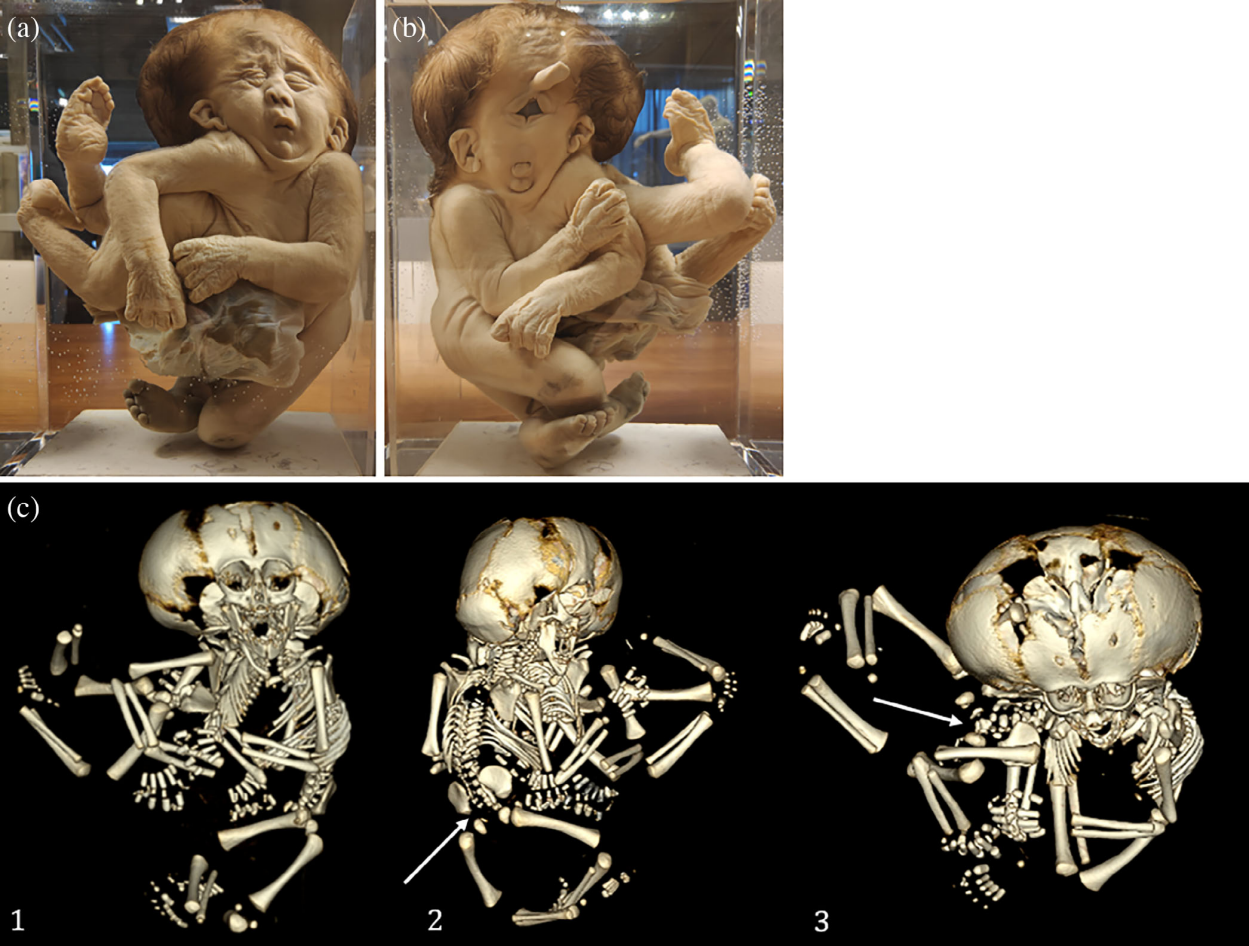
(a) and (b) “ventral“ and “dorsal” views of cephalothoracoileopagus twins with externally visible omphalocele and (although unnoticeable in the photograph) both cloacal exstrophy and imperforate anus. Furthermore, the retro‐position and club feet of the lower limbs and profound kyphoscoliosis result from the spinal defect, which is often seen in severe cases of OEIS complex. Note the holoprosencephalic appearance and proboscis (blind‐ending elongated appendage)‐like structure above the affected eye. (c) Three‐dimensional reconstructed skeleton images based on computed tomography (CT) data of the specimen in which severe spinal defects in both twins are observable (white arrows). Specimen from the Anatomical Museum in Nijmegen (The Netherlands)

**FIGURE 10:**
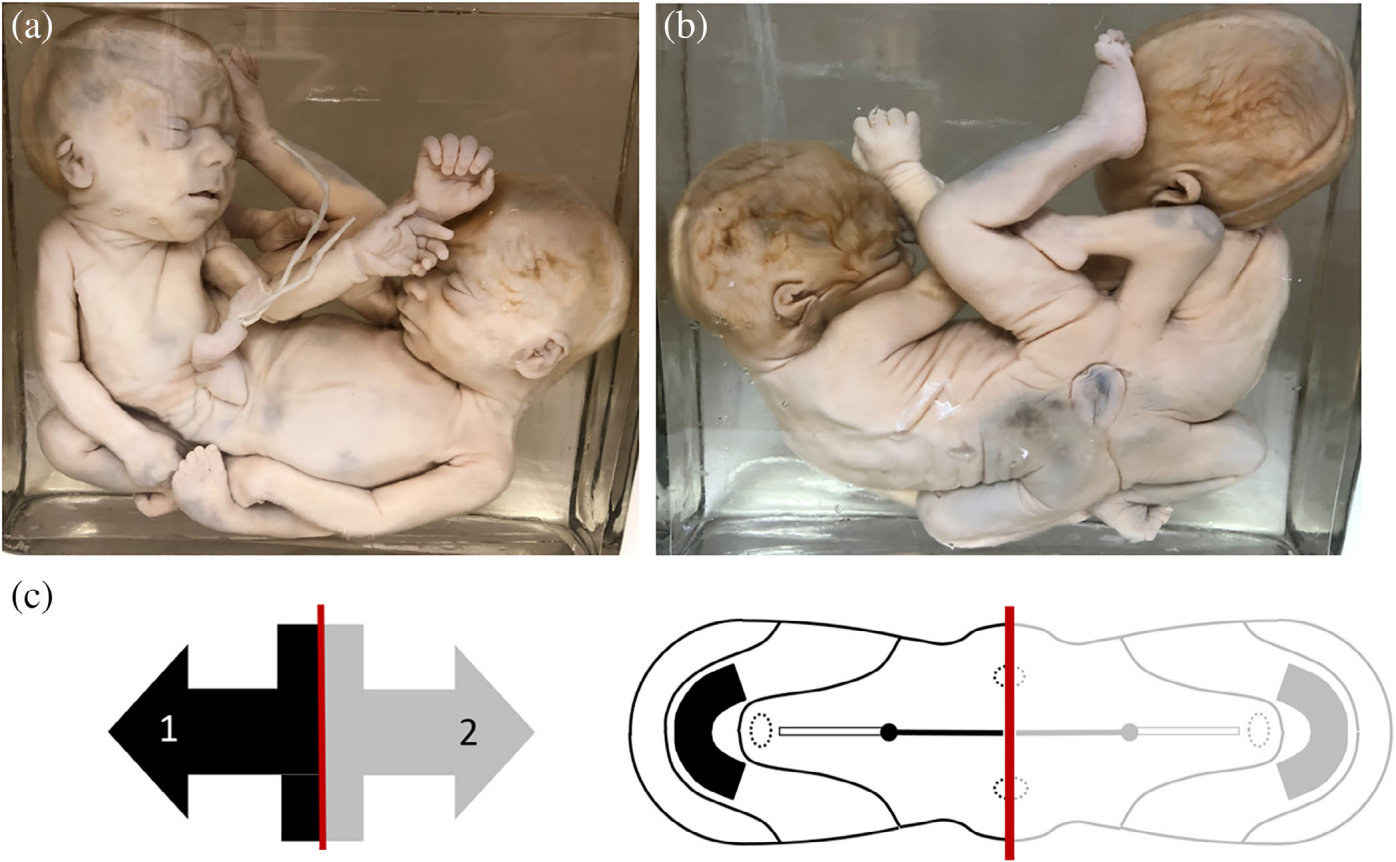
(a) and (b) Ventral and dorsal views of an ileoischiopagus tetrapus with a somewhat broadened umbilical base. Specimen from the *Narrenturm* collection in Vienna (Austria). (c) Schematic representation of the process when neo‐axial orientation sets in within caudal conjunctions, indicated by black (fetus 1) and gray (fetus 2) arrows. This embryological premise will produce caudally‐located structures that are being divided and diverted. This configuration is further delineated in a schematic embryonic disk from the dorsal aspect of an ileoischiopagus. The red line indicates the midline of the twins, clearly showing a 50% portion of each twin in caudal areas. A minimum lateral deviation from the midline is seen within the configuration of the lower limbs, located in the cranial direction of the twins (not shown in the embryonic disk). Again, note the broad circumferential umbilical ring (the outer contour of the embryonic disk), which is paramount in the formation of omphaloceles in twins united in this manner

Both ventrally and caudally united twins are considered to be formed by duplications of primordia located more or less opposite. These duplications are influenced by neo‐axial orientation and potentially interfere in morphogenetic pathways and the ultimate morphological fate of structures close to the embryonic umbilical ring. The embryonic disk configuration in both groups inherently shows a wide circumferential umbilical ring and creates a situation favorable for forming ventral body wall defects such as omphalocele and OEIS complex (Boer et al., 2019). Ventrally united twins (e.g., omphalopagi, thoracoileopagi, and cephalothoracoileopagi twins) often show an omphalocele shared by both twin members (Austin, Schifrin, Pomerance, Gans, & Komaiko, 1980; Ornoy, Navot, Menashi, Laufer, & Chemke, 1980; Ozkur et al., 2006) (Figure 8). This omphalocele can occur in isolation or as part of a particular set of anomalies known as the OEIS complex (Oostra et al., 1998; Spencer, 2003) (Figure 9). However, this particular set of conditions is probably underrepresented in the literature as it can pass unnoticed without detailed physical examination. In contrast to their notable prevalence in ventrally united twins, shared omphalocele or OEIS complexes have rarely been reported in caudally united ileoischiopagus twins (Khan, 2011; Spencer, 1996, 2003) (Figure 10).

In singletons, the etiopathogenesis of omphaloceles is still debated and causes are sought in aberrant formation and development of the ventral body wall, deviant formation of the amnion and/or the umbilical ring, or stagnant retractions of midgut structures (F. A. Khan, Hashmi, & Islam, 2019). In addition, malfunction of embryonic folding process due to defective cell–cell signaling at the critical transition point between the amnion and the ventral body wall or between the lateral and ventral body walls at the umbilical ring could interfere with ventral body wall formation, leading to separation of the left and right rectus abdominis and accompanying fascia (Brewer & Williams, 2004). The etiopathogenesis of the OEIS complex is also debated; it could concern defective septation of the cloaca or failure of cloacal membrane breakdown, both with additional abnormalities of the adjacent lumbosacral somites (Smith, Chambers, Furness, & Haan, 1992). Alternatively, defects of caudally located mesodermal lineages are held responsible for the OEIS phenotype (Lee et al., 1999). The much higher incidence of omphaloceles and OEIS complexes in ventrally united twins than in singleton cases suggests that these conditions are caused by mechanical disturbances in the broadened circumferential umbilical ring. It is conceivable that because the body wall arrangement is duplicated and has to close over a much wider area than normal, the arrested progression of the amnio‐ectodermal junction toward the umbilicus persists. This avascular and overarching amniotic lining would then fail to function in the migration of mesodermal lineages, disrupting the formation of the ventral body wall and caudal region, subsequently resulting in a shared omphalocele/OEIS complex. In addition, retraction of opposing midgut structures inside the abdominal cavities of both twins during subsequent development could be rendered mechanically impossible. As a result, these shared structures will remain in the conjunction plane, forming the content of an omphalocele. Understandably, and to the best of our knowledge, laterally united twins with concomitant omphaloceles or cloacal exstrophy have not been reported to date: as a result of the interaction aplasia, these twins share an almost singular abdominal cavity and umbilical ring in which retraction of midgut structures is not mechanically hindered.

## 7 NEURAL TUBE DEFECTS IN PARAPAGUS AND CEPHALOTHORACOILEOPAGUS TWINS

**FIGURE 11:**
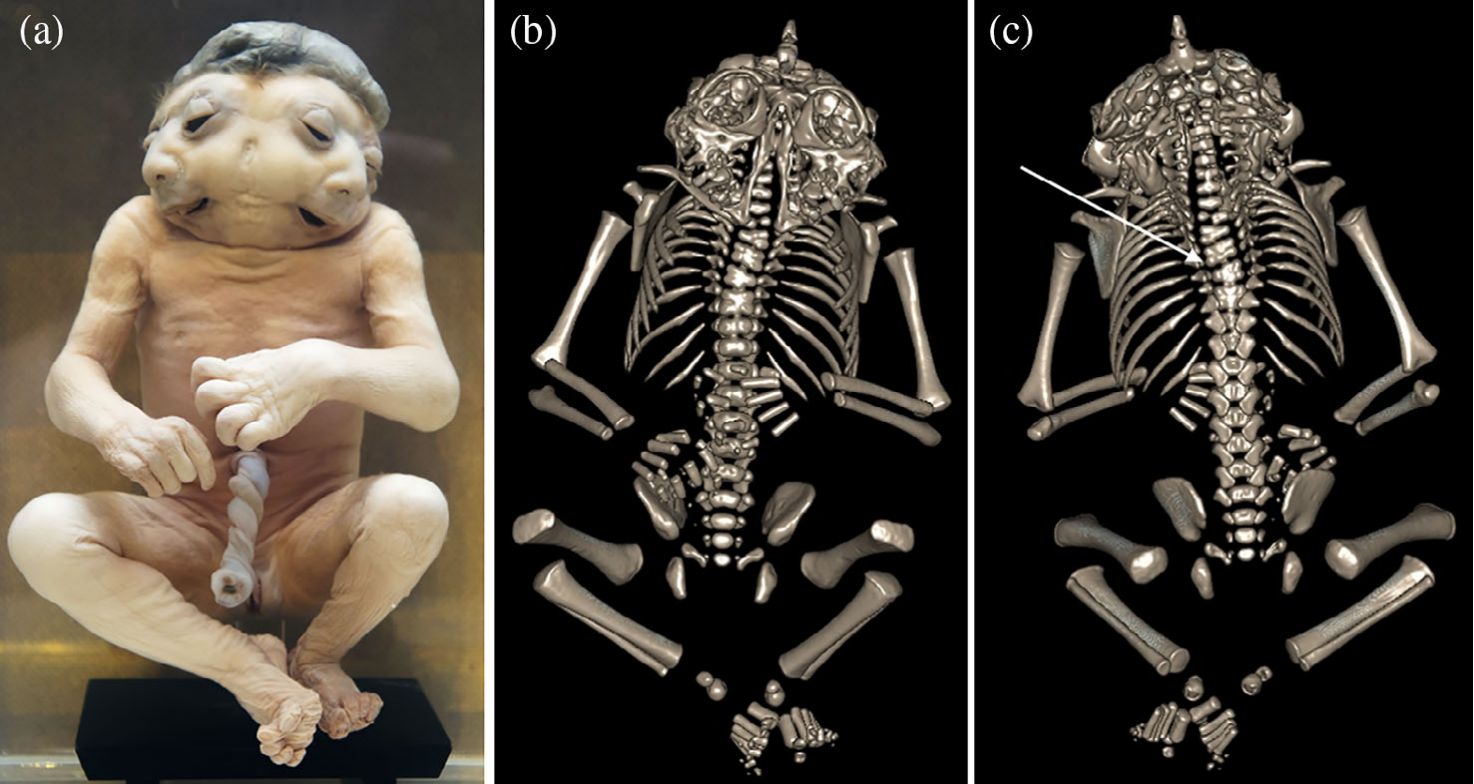
(a) Ventral view of a parapagus diprosopus with a shared craniorachischisis. Specimen from the Anatomical Museum in Nijmegen (The Netherlands). B/C. Three‐dimensional reconstructed skeleton images based on computed tomography (CT) data of the conjoined twins depicted in panel (a), in which both the cranial (b) and extensive neural tube defect on the back (white arrow) can be appreciated (c)

**FIGURE 12:**
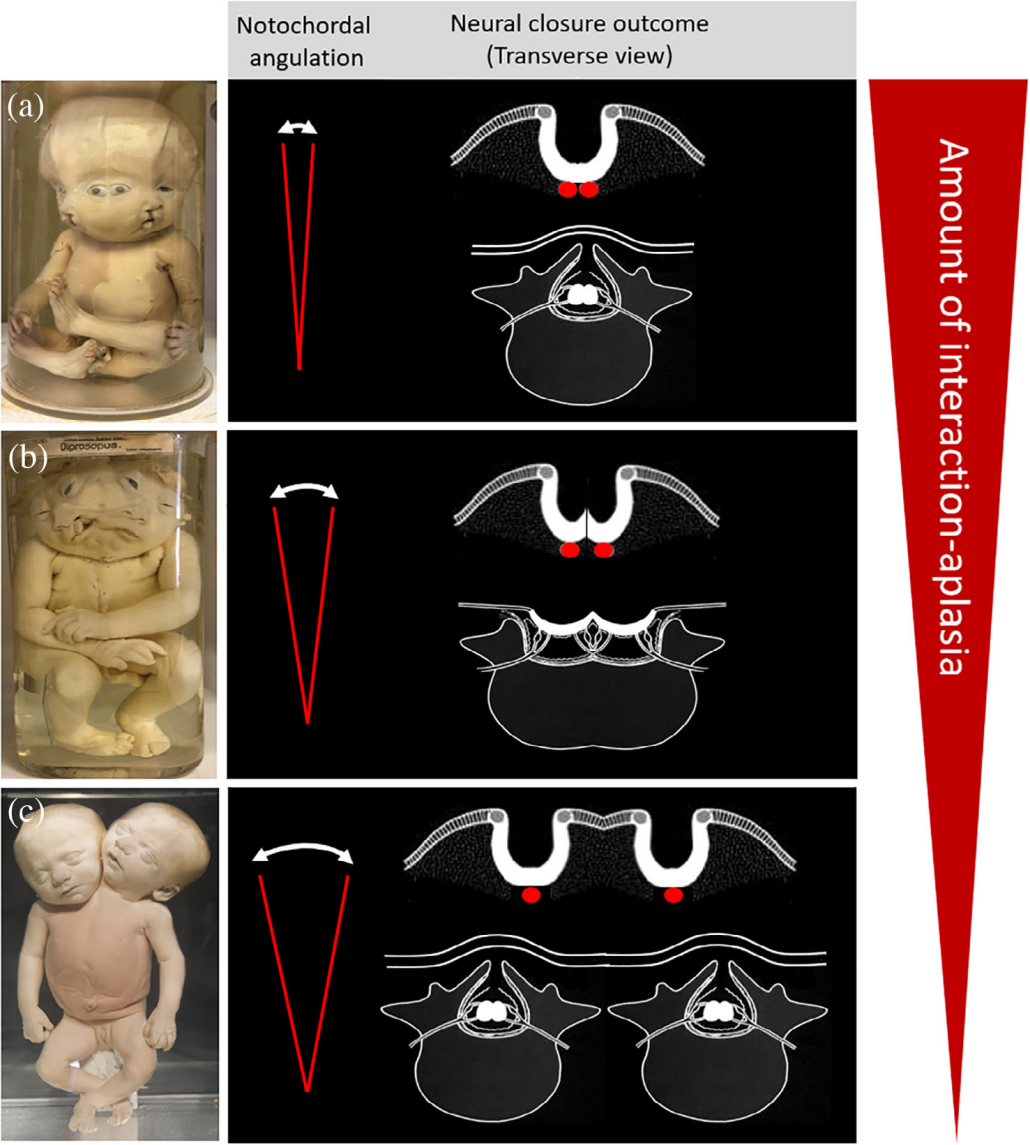
Schematic representation of different angulated configurations of notochordal duplications in parapagus twins with different degrees of interaction aplasia leading to specific dysmorphological phenotypes (red spectral triangle on the right): severe interaction aplasia (acutely approximated duplicated primordia) ultimately creates phenotypes with less medially‐located structures. (a) Parapagus diprosopus twin with acutely angulated and hence closely‐approximated notochordal duplications, implying a profound area affected by interaction aplasia. However, two neural folds (one for each twin) are located in such an approximate configuration that they are still capable of closing. Specimen from the *Narrenturm* collection in Vienna (Austria). (b) Parapagus diprosopus in which there is less acute angulation and hence a greater mutual distance between the notochordal duplications. This configuration leads to a shared craniorachischisis because the two lateral neural folds are too distant from each other to close entirely. Specimen from the *Narrenturm* collection in Vienna (Austria). Note the additional discordant cleft lip/palate in one of the twin members. (c) Parapagus dicephalus in which the mutual distance of the duplicated notochords allows two complete sets of neural folds to develop, implying the formation of two complete and closed neural tubes and their subsequent structures. Specimen from the Anatomical Museum in Nijmegen (The Netherlands)

Parapagus diprosopus twins are characterized by a latero‐lateral conjunction that is profoundly influenced by interaction aplasia, suggesting a single body arrangement with one or two heads and two (partial) faces (Oostra et al., 1998). However, even the most extreme forms have two (partial) vertebral columns, indicating early duplication of the notochord (Boer et al., 2019). Diprosopi often feature a shared neural tube defect such as anencephaly with or without craniorachischisis (Bidondo et al., 2016; Chikkannaiah, Prathap, & Venkataramanappa, 2018) (Figure 11a,b). The presence or absence of neural tube defects in parapagus conjoined twinning appears to correlate with the notochordal angulation and hence with the degree of interaction aplasia. Because of the near‐parallel position of the longitudinal axes, both medial body halves, including the medial halves of the two neural grooves, are almost completely absent owing to interaction aplasia. As a result, the remaining parts of the neural folds approximate each other to such a degree that closure of the neural tube is not compromised (Figure 12a). In the event of increased angulation between the longitudinal axes, the remaining parts of the neural folds can be too widely spaced to overarch the neural groove completely, resulting in closure defects (Figure 12b). However, when the angle between the longitudinal axes is even more obtuse, as in parapagus dicephalus, the medial halves of the neural grooves form normally, leading to two separate neural tubes without closure defects (Figure 12c).

**FIGURE 13:**
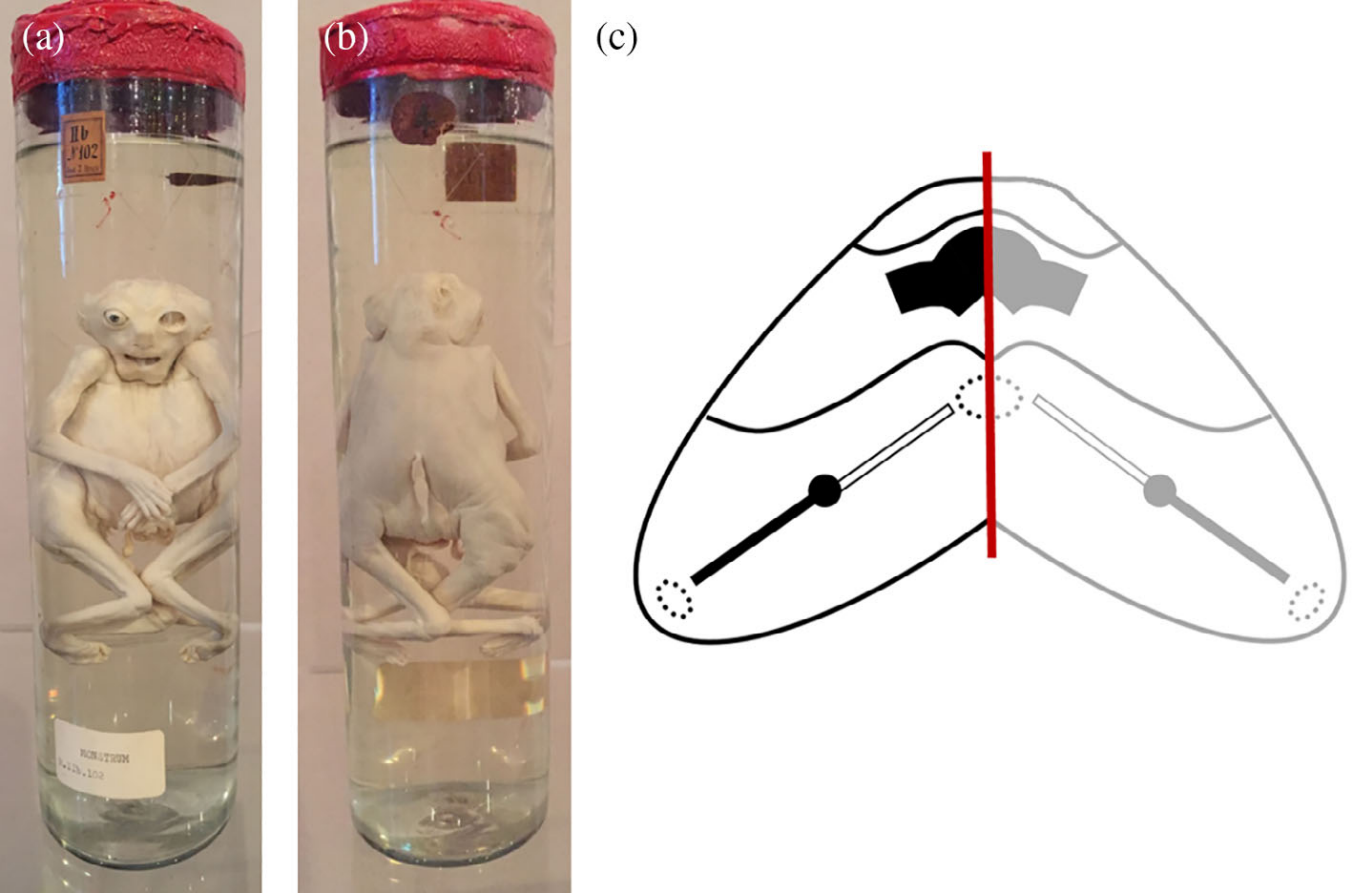
(a) and (b) Ventral and dorsal views of a cephalothoracoileopagus dibrachius tetrapus with extreme laterally deviating (cranial) ends, resulting in the absence of two of the four upper limbs, absence of a compound face on the posterior side, and shared anencephaly. Note the presence of two chins and a very broad mouth, indicating that this specimen can be interpreted as affected by over‐lateralization and showing characteristics of both ventrally (cephalothoracoileopagus) and laterally (parapagus diprosopus) conjoined twinning, but markedly different from what is seen in, for example, thoracoileioischiopagus (see Figure 5). This phenotype is extremely rare and, to the best of our knowledge, has previously been described only once (Gunter, 1946). Specimen from the Anatomical museum *Bleulandinum* in Utrecht (The Netherlands). (c) Embryonic disk configuration resembling the gross anatomy depicted in panels (a) and (b)

In addition to diprosopus, cephalothoracoileopagus twins with extreme laterally deviating cranial ends of their longitudinal axes, inducing interaction aplasia in cranially located structures, can have a shared anencephaly (Zhang et al., 2014) or encephalocele (Ozkur et al., 2006) (Figure 13). The cranial configuration of near‐parallel neural folds is comparable to that in the parapagus types described above and can therefore result in a shared neural tube defect. It is noteworthy that anencephaly and encephalocele, both closure defects of the rostral neuropore, are exclusively seen in cephalothoracoileopagus twins with profound interaction aplasia at the cranial end due to deviating cranial axes. Here, either the compound faces are both in a “ventral” position rather than on the opposing “ventral” and “dorsal” aspects of the twins (Sperber & Machin, 1987), or the “dorsal” compound face is extremely hypoplastic. As in diprosopus, the near‐parallel configuration of interaction aplasia‐affected neural folds creates an ideal situation for mechanical closure failure (Machin, 1993; Sperber & Machin, 1987).

## 8 DISCUSSION AND CONCLUSIONS

The shared anomalies discussed in this paper are among the most frequently‐occurring in conjoined twins, or are even obligatory. Nevertheless, they especially concern externally recognizable conditions; a compulsory choice, as we deal with specimens of museological value, only to be investigated by non‐invasive techniques; which in practice mostly comes down to external inspection. Although this is an indirect approach, the external dysmorphological aspects can be seen as a gateway to (or mirror of) internal characteristics, as the two are closely intertwined. We were able to pair these findings with imaging data in at least some cases, but we realize that much information on internal morphology is lacking. The potential need for this information to diagnose the encountered conditions adequately is demonstrated dramatically in, for example, identifying the nature of the “holoprosencephaloid” anomalies seen in the posterior compound face of cephalothoracoileopagus twins. Despite the external resemblance, imaging of the brain in these cases does not confirm the presumed holoprosencephaly (Winter, Kennedy, & Woodward, 2015).

The question remains: to what extent can shared anomalies in conjoined twins be compared to similar conditions in singleton cases, especially with respect to their alleged etiology and pathogenesis? In conjoined twins, as we have argued here, these anomalies result mostly if not entirely from the twinning mechanism itself and the inherently associated processes of neo‐axial orientation and interaction aplasia. Of course, these mechanisms are absent (or are still unknown or seen as unrelated) in singleton cases, yet their presenting phenotypes are strikingly similar, implying that whatever the causes in singletons, they could act along highly comparable pathogenetic trajectories. Thus, we believe it is likely that mechanical factors lead to the defects in Siamese twins. By analogy, similar defects in singletons could also be caused by mechanical factors, induced by whatever influences. However, these hypotheses need further investigation, for example, by genetic research on conjoined twins, to find answers in a possible genetic background to the etiopathogenesis of these twins: a field that has not been explored thoroughly. In our opinion, this realization offers opportunities not only to shed more light on the mechanisms involved in the development of these anomalies, both in singletons and in conjoined twins, but also on the enigmas of conjoined twinning itself. Unfortunately, new clinical cases (although very rare) are nowadays somewhat “neglected” as they are almost inherently *infaust*, have little risk for recurrence and are (oddly enough) not within the scope of additional (genetic) research. Complementary diagnostics and subsequent interpretations are therefore lacking, leaving the etiology and morphogenesis of conjoined twins hidden behind a multitude of questions. Striving to find answers to these questions requires first and foremost a meticulous inventory of all sequelae by inspection, imaging and examination of forthcoming cases of conjoined twinning. These clinical curiosities should be evaluated and described accurately by all available means.

## CONFLICT OF INTEREST

The authors declare no potential conflict of interest.
